# Hepatitis C Genotypes in Hemophilia Patients in Iran 

**Published:** 2011-02-01

**Authors:** F Bokharaei-Salim

**Affiliations:** 1 Department of Virology, Tehran University of Medical Sciences, Tehran, Iran

**Keywords:** Hepatitis C, Genotype, Hemophilia, Iran

Dear Editor,

I genuinely enjoyed reading the excellent and informative original article by Keshvari and colleagues in your journal recently.[1] Patients with hemophilia are at greater risk for all forms of viral hepatitis, especially hepatitis C than other patients.[2] The prevalence of hepatitis C virus (HCV) infection in Iranian patients with hemophilia was from 15.6% in Fars to 76.7% in North-West of Iran.[3] Recently, a meta-analysis was done by Alavian et al. and demonstrated that the prevalence rate of HCV infection among Iranian hemophilic patients was almost 40.8%.[4] The HCV genotypes and subtypes should be determined before starting any treatment for HCV infection, as it determines the selection of a proper antiviral therapy, and the duration of treatment too.[5]

According to the Keshvari et al. article,[1] the most frequent HCV genotype was 1a (58%), followed by genotype 3a (18.5%), genotype 1b (14.7%), and mixed HCV genotypes were detected in 6.2% of cases. These findings were compatible with previous studies in Iran.[6][7][8] The pattern of HCV genotypes in their patients was similar to Western Europe and different to Middle East countries, thus the author denotes to this pattern of HCV genotype in Iran due to probable use of clotting factors from Europe before 1987.

In their study, there was no association between HCV genotypes and viral load, liver biochemical profiles, and splenomegaly although these findings were corresponded to other studies about this association. The sample size of study was high (367 hemophilic patients with clinically and laboratory proven chronic HCV infection), and complete information on subjects were reported including bleeding disorder types, demographic parameters, biochemical profile that were well summarized in the tables.

There are some problems in design of some tables such as Table 1. It would be better that the title of each category (Bleeding disorder type, Bleeding severity, Laboratory parameters, HCV genotypes, Splenomegaly) to be distinguishable from their subcategory, but regarding Table 2 nd 3, they were good organized. However, these small problems cannot reduce the value of this article. As we evaluated the HCV genotypes in 8759 Iranian HCV infected patients (In Press), showing that subtype 1a was the most frequent one in patients less than 40 years (39% vs. 36.4%) and subtype 3a was the most frequent in patients older than 40 years (33% vs. 29.1%). It is better to analyze the HCV genotypes in hemophilia patients while considering the age too. Finally, I appreciate this absolutely perfect and informative article on genotyping of HCV in Iranian patients with hemophilia that can enhance and improve our knowledge about this disease in our country.
